# A novel rapid bioluminescence-based antimicrobial susceptibility testing method based on adenosine triphosphate consumption

**DOI:** 10.3389/fmicb.2024.1357680

**Published:** 2024-02-09

**Authors:** Elif Arik Sever, Esma Aybakan, Yeşim Beşli, Onur Karatuna, Tanil Kocagoz

**Affiliations:** ^1^Department of Medical Biotechnology, Institute of Health Sciences, Acibadem Mehmet Ali Aydinlar University, Istanbul, Türkiye; ^2^Department of Biostatistics and Bioinformatics, Institute of Health Sciences, Acibadem Mehmet Ali Aydinlar University, Istanbul, Türkiye; ^3^American Hospital Clinical Laboratory, Istanbul, Türkiye; ^4^EUCAST Development Laboratory, Clinical Microbiology, Central Hospital, Växjö, Sweden; ^5^Department of Medical Microbiology, School of Medicine, Acibadem Mehmet Ali Aydinlar University, Istanbul, Türkiye

**Keywords:** antimicrobial susceptibility, bioluminescence, adenosine triphosphate, antibiotics, beta-lactam

## Abstract

**Introduction:**

Standard, phenotypic antimicrobial susceptibility testing (AST) methods require 16–20 h of incubation and are considered as the bottleneck in providing timely input for appropriate antimicrobial treatment. In this study, a novel adenosine triphosphate (ATP)-bioluminescence-based method which allows rapid AST within 3 h was described.

**Methods:**

Standard AST was performed for 56 *Enterobacterales* isolates using EUCAST disk diffusion (DD) methodology. For the bioluminescence-based rapid AST, suspensions of bacteria were prepared using Mueller–Hinton broth to obtain a turbidity of 0.5 McFarland. The suspensions were distributed into 96-well microtiter plates. ATP (20 mM) and fixed concentrations of different antibiotics were added. Following incubation at 37°C for 1 h, a luminescent reaction mixture, including the substrate luciferin and luciferase enzyme solutions, was added. The chemiluminescence was monitored using an imaging system. Light production demonstrated the presence of ATP, indicating that the isolate was susceptible to the antibiotic in the well. Absence or decrease of light intensity, compared with the growth control well, indicated the use of ATP as an indirect measure of bacterial growth, and therefore resistance to the antibiotic in the well.

**Results:**

The novel AST method was tested using a total of 348 test wells. Concordance was achieved for 290 (83.3%) of the tests, whereas 52 (14.9%) and 6 (1.7%) tests caused minor and major errors, respectively.

**Discussion:**

In this study, a bioluminescence-based rapid AST was developed based on the consumption of ATP by bacteria. Our method’s uniqueness relies on determining ATP consumption by microorganisms in the presence or absence of an antibiotic. The novel AST method described in this study lays the groundwork for obtaining rapid results, which should be considered as a proof of concept. With further optimization studies, this novel method can provide higher accuracy and be introduced into clinical practice as a routine AST method.

## 1 Introduction

Antimicrobial resistance poses a threat to global public health as it costs lives and undermines the success of modern medicine. Considering the increasing antimicrobial resistance, the success of effective treatment of bacterial infections relies on the selection of appropriate drugs. This has increased the need for reliable antimicrobial susceptibility test (AST) results from clinical microbiology laboratories. Current phenotypic AST methods require a long incubation period, usually 16 to 20 h. However, for certain bug-drug combinations, such as *Staphylococcus* spp. and vancomycin, an extended incubation of 24 h is required [Clinical and Laboratory Standards Institute (CLSI), 2021; European Committee on Antimicrobial Susceptibility Testing (EUCAST, 2023a,b)]. This delays personalized treatment for patients and gives microorganisms the opportunity to develop resistance to empirically used antibiotic agents, which often include broad-spectrum antibiotics.

To address the need for rapid AST results, many new techniques have been recently developed (van Belkum et al., 2020). Several of the recently developed techniques focus on patients with sepsis and enable direct AST from positive blood cultures (e.g., Accelerate PhenoTest^®^ BC Kit, Accelerate Diagnostics; ASTar, Q-linea; dRAST, Quantamatrix; QuickMIC, Gradientech; and Specific Reveal, bioMérieux). The primary obstacles for these systems are the high cost of consumables, low capacity of the instruments to run parallel tests, and the limitation of being able to test only for the available antibiotics present in the kit. EUCAST and CLSI have recently developed methods to shorten the incubation time required for quicker AST results. EUCAST has developed the “rapid AST from blood culture bottles (RAST)” method, which enables disk diffusion performed directly from positive blood cultures. This method is optimized to read the AST results at 4–8 h. If the zone diameters remain unreadable after 8 h, incubation is extended to standard 16–20 h. The RAST method has been validated for *Escherichia coli*, *Klebsiella pneumoniae*, *Pseudomonas aeruginosa*, *Staphylococcus aureus*, *Streptococcus pneumoniae*, *Enterococcus faecalis*, *E. faecium*, and *Acinetobacter baumannii* with some relevant antimicrobial agents (Åkerlund et al., 2020). CLSI has also described a similar methodology; however, CLSI’s method is currently limited to *E. coli* and *P. aeruginosa* only. CLSI recommends an early reading at 8–10 h, which is extendable to standard 16–18 h if unsatisfactory growth is achieved. The new rapid AST methods described by EUCAST and CLSI drastically decrease the turnaround time for blood cultures but fail to meet some critical needs, such as susceptibility to colistin in gram-negative organisms that cannot be tested with the disk diffusion method.

The development of a universal AST method has long attracted researchers to use adenosine triphosphate (ATP) as an indicator of metabolic activity (growth or inhibition of growth in the presence of an antibiotic to determine susceptibility) (Thore et al., 1977; Wheat et al., 1988, 1989; Limb et al., 1991).

Although much has been achieved in the field of rapid AST, there is an ongoing need for a cost-effective and versatile method that can be routinely used for as many bug-drug combinations as possible. To address this need, a proof of principle study has been conducted and the suitability of the bioluminescence-based rapid AST method has been assessed. It was shown that AST results are obtainable within 3 h. The versatility of this novel technique makes it a good candidate for rapid AST, which is applicable to various organisms with relevant antibiotics. This study aimed to (i) demonstrate that the described technique is suitable for this purpose, i.e., enables rapid AST results, and (ii) perform a pilot study to optimize the testing conditions for a selected group of organisms (*Enterobacterales*) with a group of antibiotics (beta-lactams). It was concluded that following rigorous optimization, the method can be routinely used as a rapid AST method and provide clinicians with timely and reliable information that can improve clinical outcomes.

## 2 Materials and methods

### 2.1 Bacterial isolates

A total of 56 non-consecutive, non-duplicate clinical isolates of *Enterobacterales* (without and with suspected resistance to relevant agents) originating from human infections were selected (Table 1).

**Table 1:** Number of tested *Enterobacterales* strains.

Strains	*n*
*Escherichia coli*	29
*Proteus mirabilis*	2
*Klebsiella pneumoniae*	19
*Morganella morganii*	2
*Klebsiella oxytoca*	1
*Enterobacter aerogenes*	1

All isolates were tested with the EUCAST disk diffusion (DD) method for non-fastidious organisms (EUCAST, 2023a). For the quality control of the Mueller-Hinton agar (Merck Millipore, USA) and antimicrobial disks (Oxoid, Basingstoke, United Kingdom), *Escherichia coli* ATCC 25922 was used. The results were interpreted using EUCAST Clinical Breakpoint Tables 13.1, 2023 (EUCAST, 2023b).

### 2.2 Luciferase production

A plasmid (pSF-OXB20-Fluc) bearing a luciferease-coding gene and a kanamycin-selective gene was obtained commercially from Oxford Genetics and was transformed into the host *E. coli* BL21 cell strain using the CaCl_2_ chemical transformation technique (Dagert and Ehrlich, 1979). Following the transformation, colonies bearing the kanamycin gene were selected on a kanamycin (50 μg/mL) selective LB agar plate. The selected colonies were then inoculated overnight in LB broth media with kanamycin at 37°C and 180 rpm until the OD 600 reached 0.5–0.6 (logarithmic phase). Enzyme purification was performed as previously described (Chen et al., 1996). The culture was centrifuged at 10,000×*g* for 10 min at 4°C, and a cell pellet was obtained. The cell pellet was resuspended in 1:10 (v:v) ratio of lysis buffer (25 mM Tris-HCI pH, 7.8, 20 mM mercaptoacetic acid, 20 mM EDTA, %10 glycerol, %1 triton X-100) and the suspension was sonicated for 20 s with breaks every 2 min in six cycles on ice before ammonium sulfate fractionation.

Ammonium sulfate fractionation was conducted as previously described with centrifugation at 25,000×*g* for 30 min at 4°C (Chen et al., 1996). The luciferase-containing fractions between 45 and 65% were taken for dissolution with 1-ml TE buffer (25 mM Tris/HCL, 1 mM EDTA, pH 7.8). This crude enzyme-containing buffer was then loaded onto a column containing Sephadex 25 (GE-healthcare) resin, which was equilibrated using STE buffer (100 mM NaCl, 10 mM Tris/HCl, 1 mM EDTA, pH 7.8). After protein concentrations were measured using the BCA kit (Thermo cat#23225), the purified luciferase enzyme was eluted via centrifugation for 1 min at 735×*g* at 4°C. The identity of the isolated luciferase enzyme was demonstrated as previously described using 10% SDS-polyacrylamide gel electrophoresis (SDS-PAGE) (Song et al., 2013). To determine the optimal luciferase production duration in bacteria, transformant bacterial cells bearing the FLuc plasmid were incubated for 2, 4, 6, and 24 h. The separated luciferase protein band (62 kDa) was identified via comparison with the 66-kDa bovine serum albumin, which was used as a molecular weight control. Enzyme aliquots were stored at −80°C until use.

### 2.3 Bioluminescence-based antimicrobial susceptibility test

**Figure 1:**
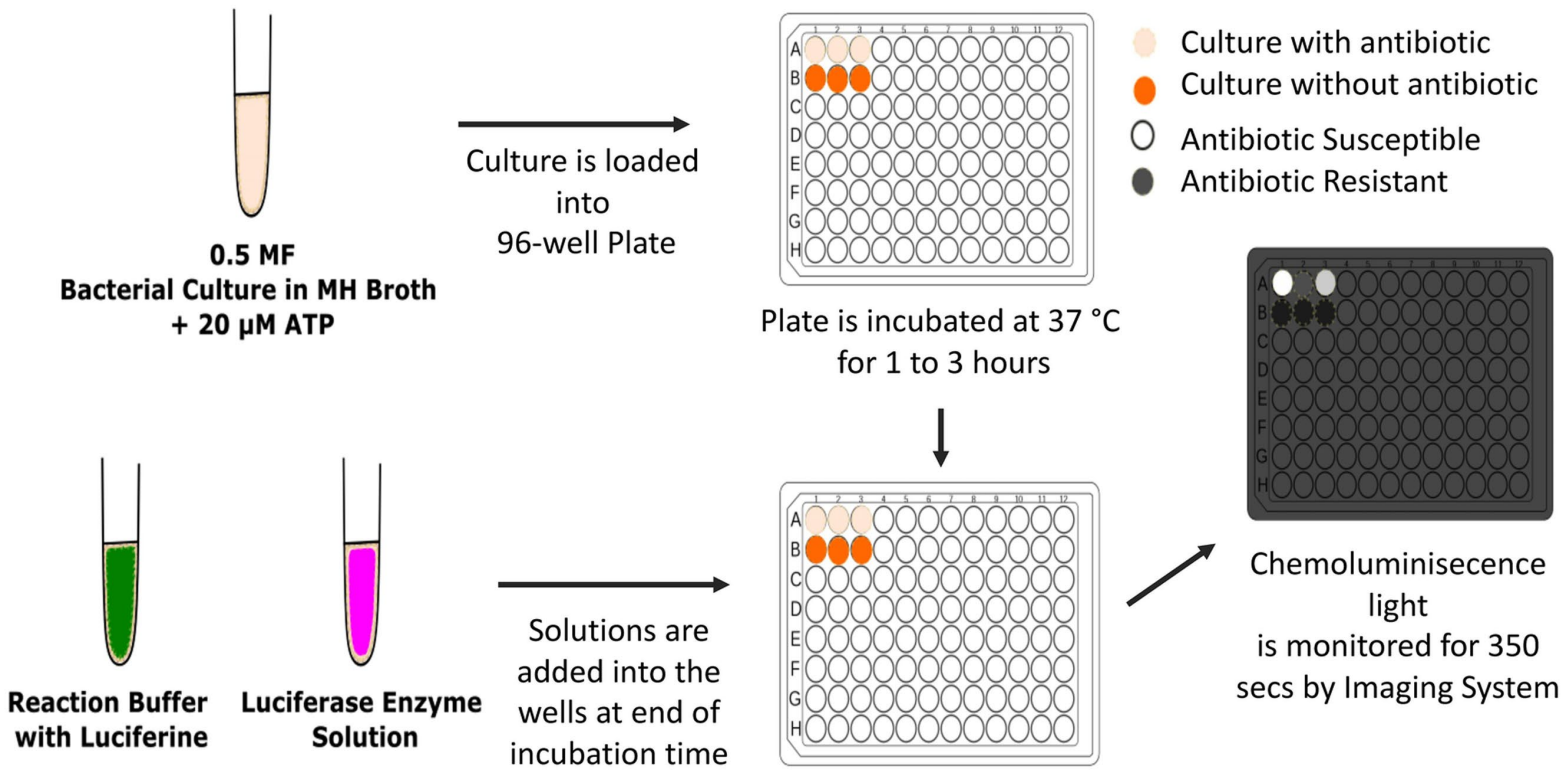
The application of bioluminescent antimicrobial susceptibility test. MF, McFarland turbidity standard; MH, Mueller Hinton. Inkscape Project. (2020). Inkscape. Retrieved from https://inkscape.org.

**Figure 2:**
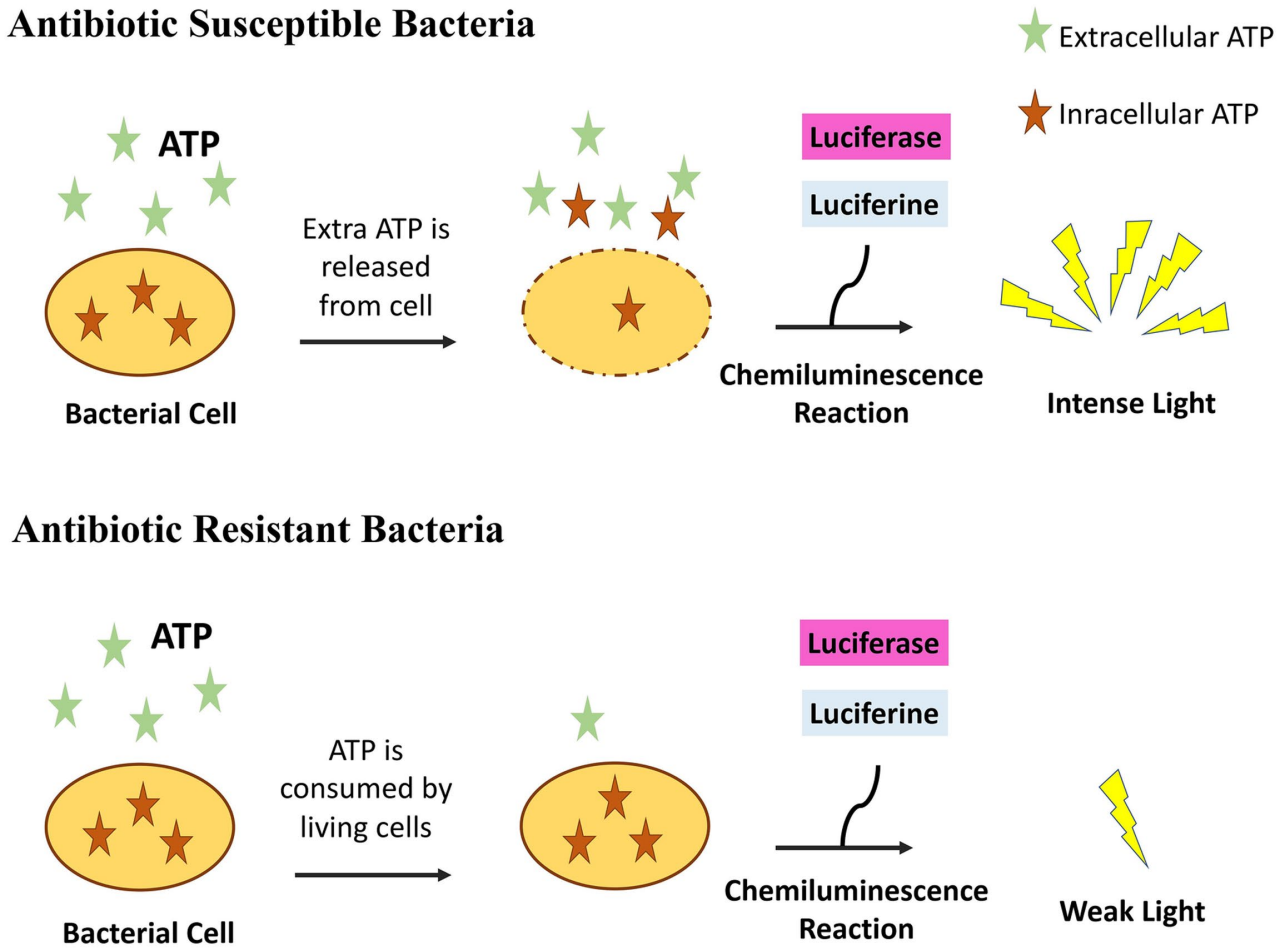
The principle of bioluminescent antimicrobial susceptibility test. If bacteria are susceptible to an antibiotic, they do not use ATP present in the medium and also bacterial ATP leaks outside increasing further ATP concentration. Light is emitted when luciferase and luciferin is added. If bacteria are resistant to an antibiotic, they grow and consume ATP resulting to the absence of light when luciferase and luciferin is added. Inkscape Project. (2020). Inkscape. Retrieved from https://inkscape.org.

Bacterial suspensions of *Enterobacterales* isolates (*n* = 56) were prepared in Mueller–Hinton broth and matched to 0.5 McFarland standard (Table 1). The suspension (50 μL) was distributed into the wells of a clear, flat-bottomed, 96-well plate. ATP (20 mM) and 10 μL of fixed concentrations of seven antibiotics (ampicillin, cefazoline, cefuroxime, ceftriaxone, cefotaxime, ceftazidime, and cefepime) were added (Table 2). Negative control well (10 μL ddH_2_O instead of antibiotic solution) and positive control well (Mueller–Hinton broth only) were included in all tests. Following incubation at 37°C for 1 h, a 50-μL reaction mixture, including the substrate 50 μM luciferin and luciferase enzyme solutions were added, and chemiluminescence was monitored using a ChemiDoc MP Imaging System (Bio-Rad, United States) (Figure 1). Light production indicated the presence of ATP, indicating that the isolate was susceptible to the antibiotic in the well. The absence or decrease in light intensity, as compared with the growth control well, indicated the use of ATP as an indirect measure of bacterial growth, and therefore resistance to the antibiotic in the well. The growth in the wells that did not show bioluminescence and inhibition in wells producing luminescence were confirmed by transferring these media to a Mueller Hinton agar plate and incubating overnight at 37°C. *Escherichia coli* ATCC 25922 was used as the susceptible quality control strain. AST results obtained using the EUCAST disk diffusion method were used to evaluate the concordance of the results obtained using the novel, bioluminescence-based, rapid AST method (Figure 2).

**Table 2:** Antibiotic breakpoint concentrations of EUCAST and novel bioluminescence-based rapid antimicrobial susceptibility test.

Antibiotics	*Enterobacterales*		Breakpoint concentrations used for bioluminescence test (mg/L)
	EUCAST MIC breakpoints (mg/L)		
	**S**≤	**R >**	
Cefazoline (CEZ)	0.001	4	2
Cefuroxime (CXM)	0.001	8	4
Ceftriaxone (CFX)	1	2	1.5
Ceftazidime (CZA)	1	4	3.125
Cefotaxime (CTX)	1	2	1.5
Ampicillin (AMP)	8	8	8
Cefepime (CFPM)	1	4	2.5

## 3 Results

### 3.1 Bioluminescence-based antimicrobial susceptibility test

**Figure 3:**
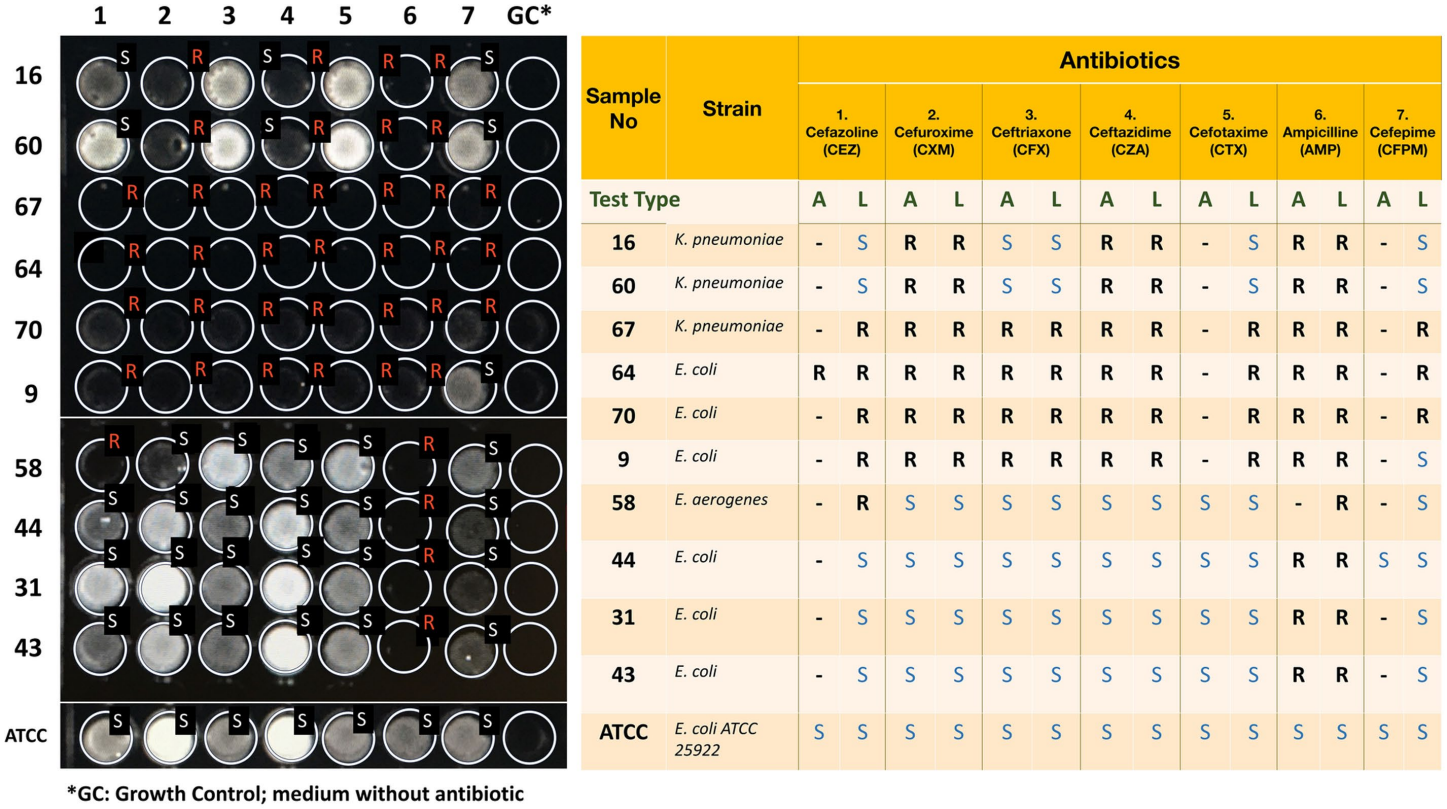
The visual results of the novel rapid bioluminescence-based antimicrobial susceptibility test (AST) and their comparison with standard AST results. Nineteen *Enterobacterales* clinical isolates and *Escherichia coli ATCC 25922*, shown in this figure, were tested using both the novel bioluminescence-based AST and standard EUCAST disk diffusion method. A stands for standard AST result obtained with EUCAST disk diffusion method, whereas L stands for luciferase-based rapid bioluminescence test. S stands for susceptible, whereas R stands for resistant. Discrepant results are highlighted. Inkscape Project. (2020). Inkscape. Retrieved from https://inkscape.org.

Fifty-six clinical strains of *Enterobacterales* were tested to determine the accuracy of the novel test (Table 1). These strains were inoculated into a total of 348 wells with antibiotics (cefazoline, cefuroxime, ceftriaxone, ceftazidime, cefotaxime, ampicillin, and cefepime) and without antibiotics. Table 2 shows the breakpoint concentration values for the different antibiotics chosen for the novel test after representative tests of trials using susceptible (S) and resistant (R) strains. The MIC values obtained using the novel bioluminescence-based rapid AST were mostly consistent with the results obtained using the standard EUCAST disk diffusion method. Two hundred and ninety (83.3%) wells were tested accurately, whereas 52 (14.9%) caused minor errors (DD AST result S, novel AST method R) and 6 (1.7%) caused major errors (DD AST result R, novel AST method S) with novel bioluminescence-based AST compared with standard DD test results. The accuracy of the test was calculated statistically as 83% (Figure 3).

### 3.2 Statistical analysis

The exact binomial test was conducted to obtain the probability of success using R Studio. The calculated number of trials was 348 and the number of successes was 290. The alternative hypothesis, which indicates a true probability of success greater than 0.5 with a 95% confidence interval, resulted in a *p*-value < 2.2 × 10^−16^. The probability of success was 83% for the binomial and accuracy tests.

## 4 Discussion

Infectious diseases are the leading cause of morbidity and mortality worldwide. The recent increase in antibiotic-resistant organisms has made it crucial to determine the antimicrobial drugs to which the infecting agent is susceptible, for the success of treatment. Most of these diseases are caused by bacteria or fungi. In these diseases, it is essential to identify the causative agent through microbiological culture and determine its susceptibility to antimicrobials. Classical antimicrobial tests require 18–24 h for rapidly growing organisms after they have been isolated in culture. The time required for slow-growing bacteria such as *Mycobacterium tuberculosis* is usually 7–12 days, even if rapid culture systems are used. In the last few years, the species of bacteria and fungi can now be determined within 30–60 min using matrix-assisted laser desorption ionization–time of flight-mass spectrometry (MALDI-TOF MS), after they are isolated in culture. However, it has been impossible to develop this rapid technique into a universal AST technique. Furthermore, rapid disk diffusion ASTs, such as the Quicolor agar disk diffusion technique providing results in 4–6 h, have been developed for various bacterial species (Kocagöz et al., 2006, 2007; Ercis et al., 2007). ATP-bioluminescence-based techniques have been developed for the purpose of AST, starting since the late 80s. This is because ATP is a good indicator of cell viability. Living organisms continuously produce ATP for their cellular reactions that require energy. Traditional ATP-based tests depend on intracellular ATP measurement after the bacteria have been killed. These methods have limitations, such as being standardized for a limited number of bacterial species or antibiotics (Puig-Collderram et al., 2022), based on preliminary processes of extracellular ATP elimination and cell lysis (Matsui et al., 2019), or longer test duration (Hattori et al., 1998). Previous studies attempted to determine the ATP content of bacteria in the presence of an antibiotic to understand the cessation of ATP production as an indication of death induced by this antibiotic. However, some studies have shown that the ATP content of cells increases when stress is created before the bacteria dies (Matsui et al., 2019). Therefore, when an antibiotic is applied, there is a paradoxical increase in the ATP content of bacteria in the early hours, followed by a reduction several hours later. Therefore, reliable AST results cannot be obtained within a few hours using these methods.

To address these limitations, we have employed a different approach in this study. The principle of our method depends on the comparison of ATP consumption by microorganisms incubated with and without antibiotics. For this purpose, microorganisms were incubated in microtiter plate wells in a medium containing ATP, with or without antibiotics. Although living microorganisms can consume ATP in a short time in wells where microorganisms are resistant to antibiotics, ATP concentration remains high in wells where microorganisms were killed by antibiotics. Mempin et al. revealed that, live bacteria of *E. coli* and *Salmonella* cultures depleted 10 μM ATP added in the medium, in 2 h. Their results suggested that ATP was most likely hydrolyzed or degraded by bacteria on their surface and was not transported into bacteria or used for phosphorylating bacterial components (Mempin et al., 2013). Since there is a paradoxical increase in ATP concentration in dying bacteria in the first few hours (Matsui et al., 2019; Ihssen et al., 2021), this may further increase ATP concentration in the medium. Therefore, when a luciferin and luciferase mixture was added to the wells; bioluminescence was less where microorganisms grew and more in wells where growth was inhibited by antimicrobials. An advantage of our approach is that, bacterial lysis, using detergents and other lytic agents that may interfere with luciferase enzyme activity is not required.

Previous ATP bioluminescence-based assays measured ATP levels using a luminometer without providing a visual display. In our technique, light production can be visualized using a camera, and the results can be obtained using an automated image processing device.

Among all strains tested, only one of the two *P. mirabilis* strains led to major errors for all tested antibiotics, and repetition of the test did not solve the problem. This requires further investigation to determine if this is related to a specific unknown feature of the strain. Furthermore, ceftazidime and ceftriaxone test results for two different *K. pneumonie* specimens were discordant with the standard antibiogram results. These specific problems reduced the accuracy of the novel rapid ATP-bioluminescence-based method to 83%. To determine if the overall performance of the test can be increased to more than 95%, further studies to understand the problems we encountered by some species of bacteria and certain antibiotics are required. More studies including larger numbers of strains susceptible and resistant to all antibiotics are also required before the novel test can be reliably used in routine daily applications.

## 5 Limitations

First, luciferin substrate starts to decay shortly after luciferase is added to the wells, and the bioluminescence reaction ends in minutes. Accordingly, the results should be measured immediately after the luciferase enzyme is mixed. Second, production and purification of luciferase is a laborious process and the stability of the enzyme requires transportation using cold chain conditions. Additionally, each bacterial species leaks different amounts of ATP from their cell membrane into their growth environment, especially if affected by an antibiotic (Mempin et al., 2013). Lastly, the consumption rate of ATP of different species differs. This makes it challenging to optimize the ATP concentration in the medium which would work equally well in all kind of different species of bacteria.

However, the test’s rapid detection of antimicrobial susceptibility may outweigh its limitations by preventing late and incorrect antibiotic prescriptions and would ultimately save many lives and money. Therefore, it is believed that the new rapid AST system may be of high interest.

## 6 Conclusion

In conclusion, a bioluminescence-based rapid AST method has been developed to detect the antibiotic resistance of bacteria. With this procedure, the antibiotic resistance of bacteria could be detected within 3 h. This method differs from the previous ATP-bioluminescence methods in the context of measuring ATP consumption by bacteria instead of ATP production in the presence and absence of antibiotics. The technique can be improved by preparing ready-to-use test plates with lyophilized antibiotics with long-term shelf life. Additionally, the novel method can be further developed for AST of slow-growing organisms such as *Mycobacterium tuberculosis* and fungi, with which promising results have already been obtained (Kocagoz et al., 2017a,b).

## Data availability statement

The raw data supporting the conclusions of this article will be made available by the authors, without undue reservation.

## Ethics statement

The studies involving humans were approved by Acibadem University Ethical Committee ATADEK. The studies were conducted in accordance with the local legislation and institutional requirements. Written informed consent for participation in this study was provided by the participants’ legal guardians/next of kin.

## Author contributions

EAS: Data curation, Formal analysis, Investigation, Validation, Writing – original draft, Writing – review & editing. EA: Data curation, Formal analysis, Investigation, Methodology, Validation, Visualization, Writing – original draft. YB: Formal analysis, Investigation, Methodology, Validation, Writing – original draft, Writing – review & editing. OK: Supervision, Writing – original draft, Writing – review & editing. TK: Conceptualization, Methodology, Supervision, Writing – original draft, Writing – review & editing.
